# A Bayesian approach to determine the composition of heterogeneous cancer tissue

**DOI:** 10.1186/s12859-018-2062-0

**Published:** 2018-03-21

**Authors:** Ashish Katiyar, Anwoy Mohanty, Jianping Hua, Sima Chao, Rosana Lopes, Aniruddha Datta, Michael L. Bittner

**Affiliations:** 1 Department of Electrical and Computer Engineering, Texas A&M University, College Station, TX, 77843-3128 USA; 2 Division of Genetics, Department of Medicine, Brigham and Women’s Hospital and Harvard Medical School, P.O. Box 1212, Boston, MA, 02115 USA; 3 Center for Bioinformatics and Genomic Systems Engineering, TEES/Texas A&M University, College Station, TX, USA; 4 Translational Genomics Research Institute, Phoenix, AZ, USA

**Keywords:** Cancer tissue heterogeneity, Bayesian modeling, Metropolis algorithm, Kernel density estimation

## Abstract

**Background:**

Cancer Tissue Heterogeneity is an important consideration in cancer research as it can give insights into the causes and progression of cancer. It is known to play a significant role in cancer cell survival, growth and metastasis. Determining the compositional breakup of a heterogeneous cancer tissue can also help address the therapeutic challenges posed by heterogeneity. This necessitates a low cost, scalable algorithm to address the challenge of accurate estimation of the composition of a heterogeneous cancer tissue.

**Methods:**

In this paper, we propose an algorithm to tackle this problem by utilizing the data of accurate, but high cost, single cell line cell-by-cell observation methods in low cost aggregate observation method for heterogeneous cancer cell mixtures to obtain their composition in a Bayesian framework.

**Results:**

The algorithm is analyzed and validated using synthetic data and experimental data. The experimental data is obtained from mixtures of three separate human cancer cell lines, HCT116 (Colorectal carcinoma), A2058 (Melanoma) and SW480 (Colorectal carcinoma).

**Conclusion:**

The algorithm provides a low cost framework to determine the composition of heterogeneous cancer tissue which is a crucial aspect in cancer research.

## Background

Cancer tissue heterogeneity is a very important aspect in cancer research with widespread implications. It is a phenomenon observed in almost all cancers including breast cancer [1], colon cancer [2], skin cancer, etc. Some of the apparent influences of cancer tissue heterogeneity are inhibition of immune cell attacks on cancer, active construction of local blood flow to the cancer and stimulation of cancer cells’ epithelial to mesenchymal transition [3, 4]. These actions enable cancer cell survival, proliferation and metastasis. As a consequence, heterogeneity is an important aspect of precision medicine and poses therapeutic challenges. The impact of heterogeneity on therapeutics for different types of cancer is presented in [5]. It is one of the causes of acquired drug resistance [6]. Acquired drug resistance is attributed to a drug resistant subpopulation of the heterogeneous cancer tissue becoming dominant after the drug successfully kills the initial dominant subpopulation. Taking this into consideration, an approach for cancer therapy mentioned in [7] relies on sustaining a particular tumor population instead of destroying as much tumor as possible. It concentrates on maintaining a dominant ratio of chemosensitive subpopulation which suppresses the growth of chemoresistant subpopulation. As a result, the tumor does not become resistant to chemotherapy. Tracking the ratio of subpopulations over time is central to this approach of therapy. Hence determining the compositional breakup of a heterogeneous cancer tissue is an important challenge to address.

In [8] an accurate, but high cost, optical approach was suggested to determine the compositional breakup of a heterogeneous cancer tissue. In this method, all the cells in the heterogeneous tissue were imaged individually and their red, green and blue fluorescence were measured. Imaging individual cells is a complex method as it requires high resolution imaging followed by complex image processing algorithms. In the proposed algorithm, we aim to develop a mathematical framework to reduce the experimental cost by relying on aggregate observations and minimizing the need for individual cell-by-cell observations. Aggregate observations are the summation of the contribution of individual cells in a heterogeneous tissue. For a setup like in [8], aggregate observations would be the separate summations of red fluorescence due to all the cells, green fluorescence due to all the cells and blue fluorescence due to all the cells in the heterogeneous cancer tissue. This would be a much simpler observation to capture as it would not require imaging the individual cells and would just need the total fluorescence hence circumventing the need for high resolution imaging and complex image processing algorithms.

In this paper we extend the gene expression based methods presented in [9, 10] so that the aggregate optical measurements from the above described technology can be used instead of gene expression measurements in order to determine the compositional breakup of the tissue under observation. Although the experimental results are provided for an experimental setup similar to the one in [8], the algorithm, however, is generic and can take any measurable quantity as an input as long as the aggregate observation can be expressed as a summation of individual cell-by-cell contributions.

The proposed algorithm requires the expensive cell-by-cell observation of individual subpopulations only once and can then be used to determine the composition of any number of heterogeneous cancer tissues composed of those subpopulations.

## Methods

Let us assume that we need to study heterogeneous cancer tissues composed of a given set of *n* different cell lines represented as *C*=(*C*_1_,*C*_2_,…,*C*_*n*_). Let there be *m* different quantitatively measured attributes. These attributes are chosen such that they are independent and the different cell lines have dissimilar attribute profiles. The idea of the algorithm is to use the expensive cell-by-cell observation of attributes to create a database of the mean and standard deviation of the attributes for these *n* cell lines in isolation. This is only a one time process as the mean and the standard deviation of the attributes of the cell lines are assumed to remain consistent for different heterogeneous cancer tissues. This is under the assumption that the cells in a heterogeneous cancer tissue do not affect the attribute value of each other. Once this is done we can analyze any heterogeneous cancer tissue composed of any subset of these *n* cell lines by collecting only low cost aggregate attribute observations. The algorithm takes as an input the mean and standard deviation of the attributes for the cell lines from the database and the aggregate attribute observations of the heterogeneous cancer tissue and gives the compositional breakup of the heterogeneous tissue as the output.

### Parameters of the attributes

The first step of the algorithm is to profile (estimate the mean and standard deviation of the attributes) each of these *n* cell lines by making high cost cell-by-cell attribute observations for them. To do this, we measure the value of the *m* attributes for individual cells of a particular cell line. We do this separately for all the *n* different cell lines. For a particular cell line, say *i*^*th*^ cell line, these individual cell observations are considered to be the samples of the random attribute vector *E*_*i*_=(*E*_*i*1_,*E*_*i*2_,…,*E*_*im*_). The algorithm uses the sample mean and sample standard deviation as the estimate of the mean and standard deviation of the attributes.

1 $$ \hat{\mu_{ij}} = \frac{1}{p} \sum_{k=1}^{p} e_{ijk}  $$

2 $$ \hat{\sigma_{ij}} = \sqrt{\frac{\sum_{k = 1}^{p} \left(e_{ijk} - \hat{\mu_{ij}}\right)^{2}}{p-1}}  $$

where *e*_*ijk*_ are the samples of the random variable *E*_*ij*_. Let *μ* and *σ* be *n* x *m* matrices whose elements are *μ*_*ij*_ and *σ*_*ij*_, the true mean and true standard deviation of the attributes for different cell lines. It is important to have sufficiently large number of samples to arrive at an accurate estimate of the mean and standard deviation.

### Bayesian analysis of heterogeneous cancer tissue

Assume that the true composition of the heterogeneous tissue is given by *N*=(*N*_1_,*N*_2_,…,*N*_*n*_) where *N*_*i*_ represents the number of cells of cell line *C*_*i*_ in the tissue. Let the corresponding ratio be represented by *π*.

3 $$ \pi = \frac{N}{\sum_{i=1}^{n}N_{i}}  $$

Assume that the aggregate attribute vector is represented by *E*_*sum*_ which is the sum of the attributes of all the cells in the mixture. The objective of the algorithm is to take *E*_*sum*_, $\hat {\mu _{ij}}$ and $\hat {\sigma _{ij}}$ as an input for 1≤*i*≤*n*, 1≤*j*≤*m* and generate an accurate estimate of *N* and *π* represented as $\hat {N}$ and $\hat {\pi }$ respectively. In other words, the algorithm takes as an input the sum of unknown number of samples generated from *n* different random vectors with independent components and the mean and standard deviation of each component of those random vectors. From this sum, mean and standard deviations it estimates how many samples of different random vectors were added to get this sum.

**Fig. 1:**
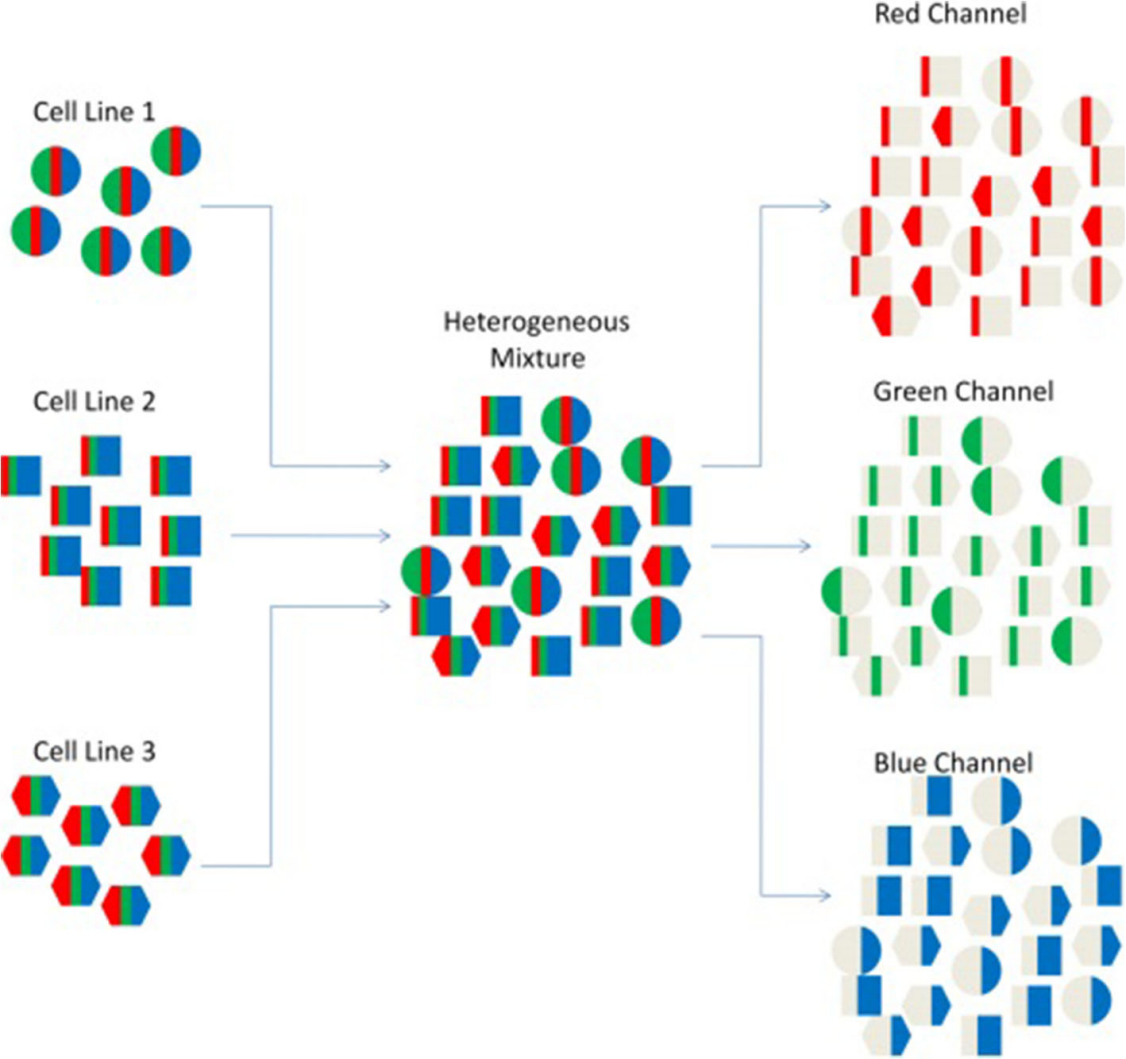
Heterogeneous cancer tissue and the attributes

Now we focus on *E*_*sum*_ which is an *m*-dimensional vector. Let the *j*^*th*^ component of *E*_*sum*_ be represented by *E*_*sumj*_ for 1≤*j*≤*m*. As a hypothetical example, let us consider the case shown in Fig. 1. In this example, the heterogeneous tissue has 3 cell lines, represented by a circle, square and hexagon. Also, the attribute vector has 3 components, red, green and blue. The aggregate attribute value of each of the red, green and blue components can be represented as the summation of the contribution of cells from cell lines 1, 2 and 3. This can be seen in the figure as each of the red, green and blue attributes of the heterogeneous cancer tissue has contributions coming from cell line 1, 2 and 3. Hence, in general, *E*_*sumj*_ can be written as:

4 $$  E_{sumj} = \sum_{i=1}^{n} E_{i sum j}, 1\leq j\leq m  $$

where *E*_*isumj*_ is the contribution of *i*^*th*^ cell line in the *j*^*th*^ attribute of the aggregate attribute vector.

There are *N*_*i*_ cells of the *i*^*th*^ cell line in the heterogeneous mixture and the summation of the *j*^*th*^ attribute of each of these cells gives *E*_*isumj*_. The *j*^*th*^ components of the attribute vector of each of these cells are independent, as the attribute value of one cell does not affect the attribute value of another cell. They are also identically distributed with the same distribution as *E*_*ij*_. Hence, by Central Limit Theorem, for sufficiently large *N*_*i*_, *E*_*isumj*_ can be approximated by a Gaussian Distribution with mean *N*_*i*_*μ*_*ij*_ and variance $N_{i}\sigma _{ij}^{2}$. There is an inherent assumption that the $\hat {\mu _{ij}}$ and $\hat {\sigma _{ij}}$ from the first step remains valid for the mixture analysis too. This calls for a precaution in experiment design. The experimental setup for the aggregate measurements needs to be the same as the one used for cell-by-cell analysis as any variation might alter the mean and standard deviation and will result in poor estimate of *N*. For practical purposes, the cell lines which form a significant part of heterogeneous cancer tissue satisfy the condition of large *N*_*i*_. Hence, *E*_*isumj*_ has a Gaussian Distribution irrespective of the distribution of *E*_*ij*_. This is a very important implication as it gives the independence of choosing any feature as a part of the attribute vector irrespective of the probability distribution of the same. The only condition is that the aggregate attribute value of the heterogeneous cancer tissue should be given by the summation of the attributes of individual cells in the tissue.

*E*_*isumj*_ for different values of *i* in Eq. 4 are independent. Hence *E*_*sumj*_ can be approximated as Gaussian with mean $\sum _{i=1}^{n} N_{i}\mu _{ij}$ and variance $\sum _{i=1}^{n} N_{i}\sigma _{ij}^{2}$. The probability of *E*_*sumj*_ can be approximated by:

5 $$  {}P(E_{sumj}|N, \mu, \sigma) \approx \\ \frac{1}{\sqrt{2\pi\left(\sum_{i=1}^{n}N_{i}\sigma_{ij}^{2}\right)}}e^{-\frac{\left(E_{sumj}-\sum_{i=1}^{n}\mu_{ij}Ni\right)^{2}}{2\sum_{i=1}^{n} N_{i}\sigma_{ij}^{2}}}  $$

As the components of *E*_*sum*_ are independent, the probability of *E*_*sum*_ is given by:

6 $$  P(E_{sum}|N, \mu, \sigma) = \prod_{j=1}^{m} P(E_{sumj}|N, \mu, \sigma)  $$

This needs to be maximized over *N* in order to obtain a maximum likelihood estimate of *N*. However the complex expression makes it difficult to solve this problem analytically. Another approach can be to evaluate the expression in Eq. 6 for different possible values of *N*. However, the complexity of the algorithm will become exponential in that case and hence it will be infeasible when the number of different cell lines is large. Hence we use a Bayesian approach to estimate *N*.

All the components of *N* are assumed to have a uniform prior from 0 to an arbitrarily large number, say *M*. The posterior probability of *N*_*i*_ is given by:

7 $$ \begin{aligned} {}P(N_{i}|E_{sum}, &N_{-i}, \mu, \sigma) \\&= \frac{P(E_{sum}|N, \mu, \sigma)P(N_{i}|N_{-i}, \mu, \sigma)}{\int P(E_{sum}|N_{i}',N_{-i}, \mu, \sigma)P(N_{i}'|N_{-i}, \mu, \sigma)dN_{i}'} \end{aligned}  $$

where *N*_−*i*_ represents all the components of *N* excluding the *i*^*th*^ component and

8 $$  P(N_{i}|N_{-i}, \mu, \sigma) = 1/M  $$

*P*(*E*_*sum*_|*N*,*μ*,*σ*) can be calculated from Eq. 6. However, evaluating the denominator term of Eq. 7 is a complex problem. This makes the problem of calculating the posterior probability of *N*_*i*_ from Eq. 7 infeasible. To address this issue, we resort to Metropolis algorithm which is a Markov chain simulation to estimate the posterior distribution [11].

#### Metropolis algorithm

The Metropolis algorithm comes in handy when it is difficult to exactly evaluate the posterior probability. In such a scenario, if it is possible to sample directly from the posterior distribution, we can generate independent identically distributed samples and use them to approximate the posterior probability distribution. However, in our case, it is not possible to sample directly from Eq. 7. To circumvent this issue we use the full conditional of *N*_*i*_ which is given by

9 $$ {}P(N_{i}|E_{sum}, N_{-i}, \mu,\sigma) \propto P(E_{sum}|N, \mu, \sigma)P(N_{i}|N_{-i}, \mu, \sigma)  $$

Suppose we have *s* samples of *N*_*i*_ from the posterior distribution in the set (*N*_*i*1_,…,*N*_*is*_). We then consider adding the proposal value $N_{i}^{*}$ which is in the vicinity of *N*_*is*_. We follow the following steps:

- 1. $N_{i}^{*}$ can be obtained by taking a sample from a symmetric proposal distribution. For eg, $N_{i}^{*}$ can be sampled from *uniform*(*N*_*is*_−*δ*,*N*_*is*_+*δ*).
- 2. Compute the acceptance ratio
$r = P(N_{i}^{*}|E_{sum}, N_{-i}, \mu,\sigma)/P(N_{is}|E_{sum}, N_{-i}, \mu,\sigma)$
- 3. Assign $N_{i(s+1)} = N_{i}^{*}$ with probability *min*(*r*,1) or *N*_*is*_ otherwise.

Substituting *P*(*E*_*sum*_|*N*,*μ*,*σ*) and *P*(*N*_*i*_|*N*_−*i*_,*μ*,*σ*) from Eqs. 6 and 8 in Eq. 9 while performing step 2, we see that *M* cancels and hence the algorithm is independent of *M*. The Markov chain formed by following the aforementioned steps has the same stationary distribution as the posterior distribution of *N*. The Markov chain needs to run for a few initial iterations before it reaches stationarity and only after that the sampling has to be done. An important consideration is the length of the neighborhood for the proposal distribution. If the neighborhood is too small, the Markov chain will take too long to reach stationarity and the samples will be too close to each other. Too large a neighborhood would result in too many samples being rejected once the Markov chain has reached stationarity. Hence the value of neighborhood parameter needs to be tuned appropriately. We draw samples from this Markov Chain after running it till it reaches stationarity. These samples are used to estimate the posterior distribution of *N*. To do this, we use a non parametric probability density function estimation, Kernel Density Estimation.

#### Kernel density estimation

Let (*N*_*i*1_,*N*_*i*2_,…,*N*_*ik*_) be the samples of the posterior distribution of *N*_*i*_ drawn from the Metropolis algorithm. The Kernel Density Estimate of the posterior distribution is given by:

10 $$ \hat{f}_{N_{i}}\left(n_{i}|E_{sum}, N_{-i}, \mu, \sigma\right) = \frac{1}{kh} \sum_{j=1}^{k} K\left(\frac{n_{i}-N_{ij}}{h}\right)  $$

Here, *K* is the Kernel function. Usually, *K* is a non-negative function with mean 0 and it integrates to 1. In our case, we will consider *K* to be standard normal.

If *K* is smooth, the density estimate obtained is also smooth which is the advantage offered by this density estimation method. An important consideration for the accuracy of density estimation is the value of the bandwidth parameter, *h*. A low value of *h* results in high variance in the estimation. A high value of *h* results in high bias in the estimation. As derived in [12], the optimal value of *h* which minimizes the squared error cost is given by:

11 $$ h_{opt} = Dk^{-1/5}  $$

where $D = \frac {R(K)^{1/5}}{\left (R\left (f''\right)\sigma _{K}^{4}\right)^{(1/5)}}$ where $R(g)=\int g^{2}(x)dx$.

Since it involves *f*, where *f* is the true posterior distribution, it is not possible to calculate the exact value of *h*. An approximation for the optimal value of *h* can be obtained assuming *f* to be Gaussian. This bandwidth is called the plug in bandwidth and is given by the expression

12 $$ \hat{h}_{plugin} = 1.06sk^{-1/5}, s^{2} = \frac{1}{k-1}\sum_{j=1}^{k}\left(N_{ij} - \bar{N_{i}}\right)^{2}  $$

Once the posterior density function estimation is done, we can evaluate the posterior mean, the posterior mode, the confidence interval, etc. Such properties of *N* can be used to come to conclusions about the composition of the heterogeneous cancer tissue. We use maximum a posteriori probability (MAP) estimate (the mode of the posterior distribution) of *N*, represented as $\hat {N}$.

### Important practical considerations

There are important factors crucial for the implementation of the proposed algorithm. The algorithm needs to know which cell lines can potentially be present in the heterogeneous mixture which is an important research problem in itself and has been widely studied. It is important to see that the algorithm does not need the exact number of different types of cell lines. Instead, it needs all the possible cell lines that might be present, that is, the cell lines considered by the algorithm can be all the cell lines that are present in the heterogeneous tissue and a few more. If any of these cell lines are not there in the heterogeneous tissue, the algorithm will estimate very low value of *N*_*i*_ for the corresponding cell line. There are a variety of methods available to study the cell lines present in a heterogeneous cancer tissue, some of which are experimental whereas others are algorithmic. Fluorescent in situ hybridization(FISH) or FISH coupled with immunofluorescence, are methods based on amplification of specific regions in the chromosome to detect heterogeneity. Another approach is to sequence genes known to be frequently mutated for the cancer under study. There have been other studies based on the study of whole genomes. A good summary of the experimental methods to detect the subpopulations of a heterogeneous tissue is provided in [4]. There have also been algorithmic approaches suggested based on clustering. There was a classification method based on the gene expression values from the Cancer Genome Atlas (TCGA) for the identification of various cell types in glioblastoma multiforme [13]. The details of these methods are beyond the scope of this paper. The important point is that these methods have been applied for different kinds of cancer and the results are available in literature, hence, such an analysis does not need to be performed for the tissue under consideration. To mention a few results, insights into breast cancer composition were provided in [14], for leukemia, the results were provided in [15], prostate cancer heterogeneity is discussed in [16], etc.

Another very crucial challenge is the sampling of heterogeneous cancer tissue. Heterogeneity is not uniformly distributed in a tumor and hence normally a single sample from the tumor is not representative of the whole tumor. In such a scenario, analysis or heterogeneity requires multiple samples from different regions of the tumor. One such example is presented in [17] where spatially separated samples of renal carcinoma are used to study intratumor heterogeneity.

## Results

### Simulated data

In order to demonstrate the performance of the proposed algorithm, we test its performance on synthetic data. We consider a 10 cell lines, 10 attribute system. We look at the effect of two parameters - the similarity of attribute mean between the cell lines and variance on the performance of the algorithm. The root square error, *e*, of estimation of *π* is used as the parameter to evaluate the performance.

13 $$  e = \sqrt{\sum_{i=1}^{n} \left(\pi_{i} - \hat{\pi_{i}}\right)^{2}}  $$

Note that it is different from the traditional root mean square error because *π* is constrained such that $\sum _{i=1}^{n} \pi _{i} = 1$ and the root mean square error would decrease as the number of cell lines increase. For the asymptotic case as *n*→*∞*, the root mean square error will approach zero irrespective of the performance of the algorithm.

We first look at the performance of the algorithm for different cases of *N*. We set *μ* to be a cyclic matrix with the first row being [100 200 300 400 500 600 700 800 900 1000]. This value of *μ* ensures that all the cell lines contribute in all the attributes, making the problem challenging, and there is a difference in the attribute mean for the different cell lines. We set the standard deviation assuming constant coefficient of variation of 1. For the cell-by-cell analysis we generate 2000 samples of each cell line from a Gaussian distribution with the corresponding mean and standard deviation. The algorithm estimates the mean, $\hat {\mu _{ij}}$ and standard deviation $\hat {\sigma _{ij}}$ from this cell-by-cell data. Next, we generate the aggregate observation *E*_*sum*_ by generating *N*_*i*_ samples for the *i*^*th*^ cell line for all the cell lines. We add the attribute values of all the samples to obtain *E*_*sum*_. Table 1 presents the results of executing the algorithm for different cases of *N*. The first case is the one where all the cell lines are present in equal proportion. The second case is when the all the cell lines are present in unequal proportions. The third case is when only half of the cell lines are actually present in the mixture. The last case is when there is only one cell line in the mixture. The third and the last case demonstrate how the algorithm can be used without knowing exactly how many cell lines are present in the heterogeneous tissue.

**Table 1:** Number of cells originally in the mixture and the number of cells estimated by the algorithm

*N* (Original)	$\hat {N}$ (Estimated)
[500 500 500 500 500 500 500 500 500 500 ]	[503 498 496 503 495 503 503 504 501 498]
[100 200 300 400 500 600 700 800 900 1000]	[90 204 302 398 498 601 702 801 895 1010]
[100 0 200 0 300 0 400 0 500 0]	[97 4 196 2 299 4 393 5 496 5]
[500 0 0 0 0 0 0 0 0 0]	[474 9 2 3 3 7 2 2 2 3]

**Fig. 2:**
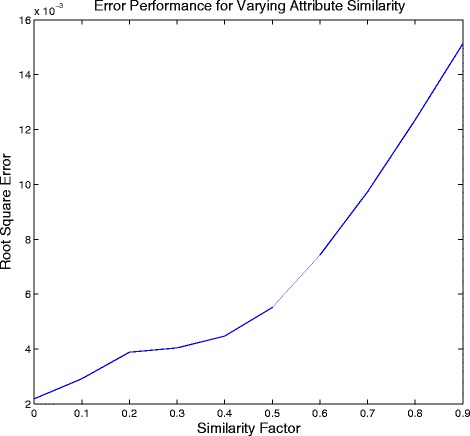
Error performance of the algorithm for varying similarity of attributes

**Fig. 3:**
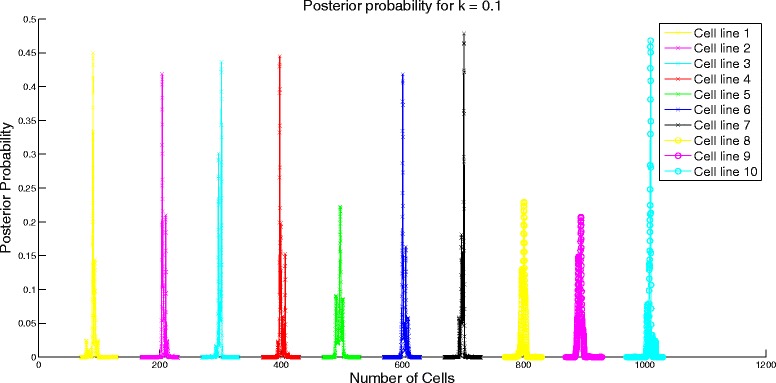
Posterior probability distribution for *k* = 0.1

**Fig. 4:**
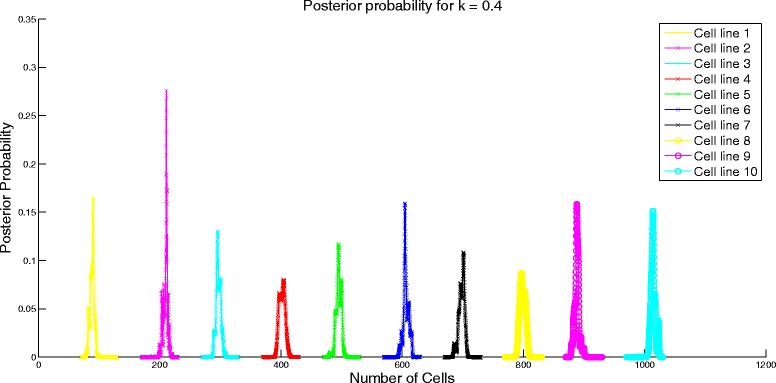
Posterior probability distribution for *k* = 0.4

Next, we analyze the performance of the algorithm by varying the similarity in the attribute means of different cell lines. We set *μ* to be a cyclic matrix with the first element of first row being 1000*k* for 0≤*k*≤1, last element being 1000 and the rest of the elements being equally spaced between 1000*k* and 1000. For instance, for *k*=0.55, the first row is [550 600 650 700 750 800 850 900 950 1000] and the rest of the rows are obtained through cyclic permutation of the first row. We set the standard deviation assuming constant coefficient of variation of 1. Similarity of the attribute means is controlled by the value of *k*. Higher value of *k* would imply more similarity of the attribute means between cell lines. When *k*=1, there would be no difference in the attribute profiles of the cell lines and it would be impossible for the algorithm to differentiate between the different cell lines. We study the effect of similarity on the error performance of the algorithm and the confidence interval. To test the algorithm, we set *N* = [100 200 300 400 500 600 700 800 900 1000]. On expected lines, the error increases as shown in Fig. 2 and the confidence interval becomes wider (evident from the change in scale of the posterior probability distribution in Figs. 3 and 4) for increasing value of *k*.

**Fig. 5:**
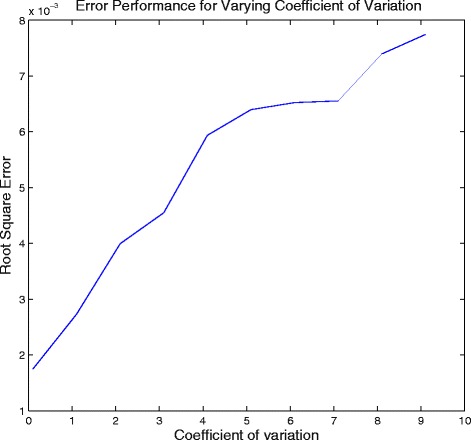
Error performance of the algorithm for varying coefficient of variation

**Fig. 6:**
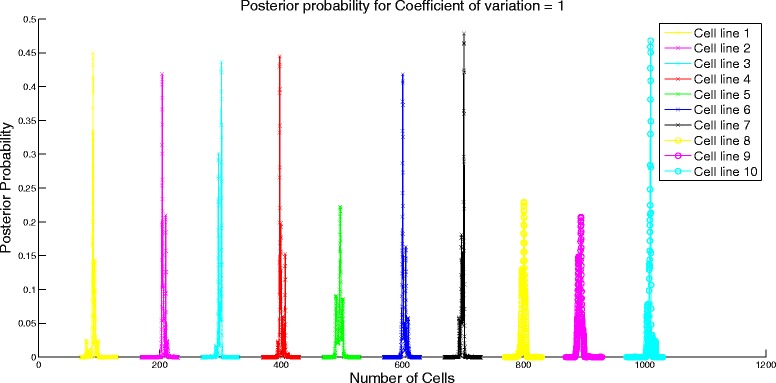
Posterior probability distribution for coefficient of variation = 1

**Fig. 7:**
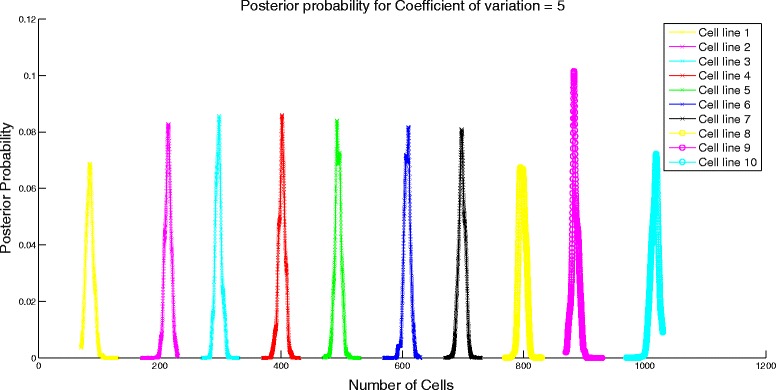
Posterior probability distribution for coefficient of variation = 5

We next analyze the effect of varying standard deviation of the attributes on the performance of the algorithm. For this analysis we set *μ* to be a cyclic matrix with the first row being [100 200 300 400 500 600 700 800 900 1000]. We also set *N* = [100 200 300 400 500 600 700 800 900 1000]. We vary the coefficient of variation to study its effect on the error performance of the algorithm and the confidence interval. As is expected, the error increases as shown in Fig. 5 and confidence interval becomes wider (evident from the change in scale of the posterior probability distribution in Figs. 6 and 7) for increase in the coefficient of variation.

### Experimental data

The algorithm was validated using the heterogeneous mixtures of three separate human cancer cell lines, HCT116 (Colorectal carcinoma), A2058 (Melanoma) and SW480 (Colorectal carcinoma). There were two different mixtures. Mixture 1 was approximately mixed in the ratio [1/3 1/3 1/3] and Mixture 2 was approximately mixed in the ratio [7/20 3/20 1/2]. Each mixture was perturbed and imaged under three different conditions: untreated, treated with Lapatinib and treated with Temsirolimus. Hence, overall there were six test cases. The experiment involved imaging the single cell lines and the mixtures on a cell-by-cell level. The attribute vector was composed of red, green and blue fluorescence. Although we have the cell-by-cell data for the mixtures too, the algorithm only takes the summation of the attribute values as the aggregate input. The cells were marked with fluorophores such that red fluorescence was emitted only by HCT116 and green fluorescence was emitted only by A2058. The blue fluorescence was used for detection of a cell and was emitted by all the three cell lines.

Note that the estimated ratio from the proposed algorithm can vary from the approximate ratio due to multiple reasons. Firstly, the estimation done by the instrument to populate the cell well is not accurate. Secondly, during the time between the cell lines being mixed and fluorescence being recorded, the cells may multiply at different rates leading to a change in the ratio. This effect might vary in the three groups due to the impact of the drugs on cell multiplication. Lastly, imaging only captures a portion of the well and it might not be a representation of the true ratio of cells in the mixture. Hence, instead of comparing the estimated ratio from the proposed aggregate observation based algorithm to the approximate ratio, we compare it to the result obtained using cell-by-cell mixture analysis algorithm proposed in [8]. Let the estimate of the number of cells obtained from [8] be $\hat {N}_{cbc}$.

**Fig. 8:**
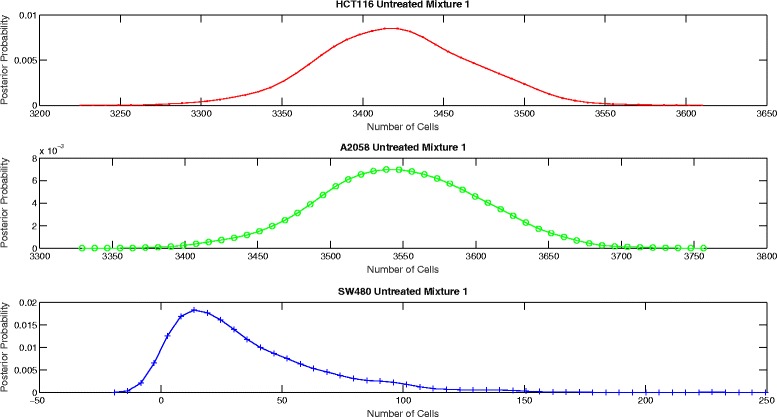
Posterior probability distribution of *N* for untreated mixture

**Fig. 9:**
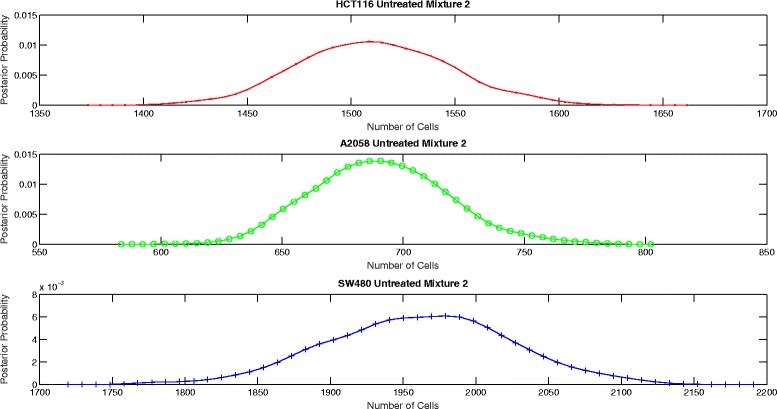
Posterior probability distribution of *N* for untreated mixture 2

**Fig. 10:**
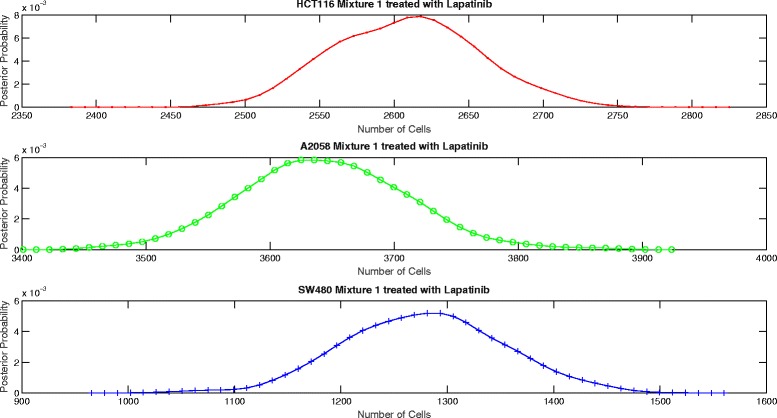
Posterior probability distribution of *N* for mixture 1 treated with Lapatinib

**Fig. 11:**
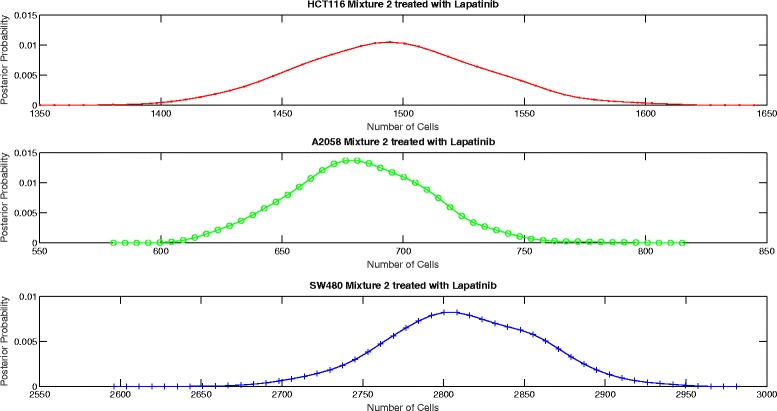
Posterior probability distribution of *N* for mixture 2 treated with Lapatinib

**Fig. 12:**
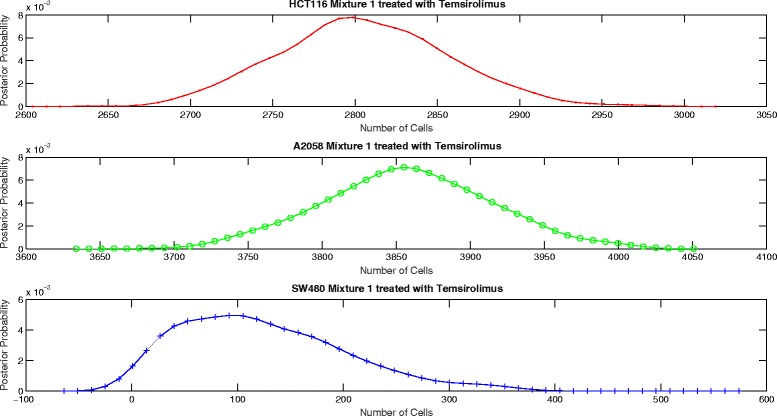
Posterior probability distribution of *N* for mixture 1 treated with Temsirolimus

**Fig. 13:**
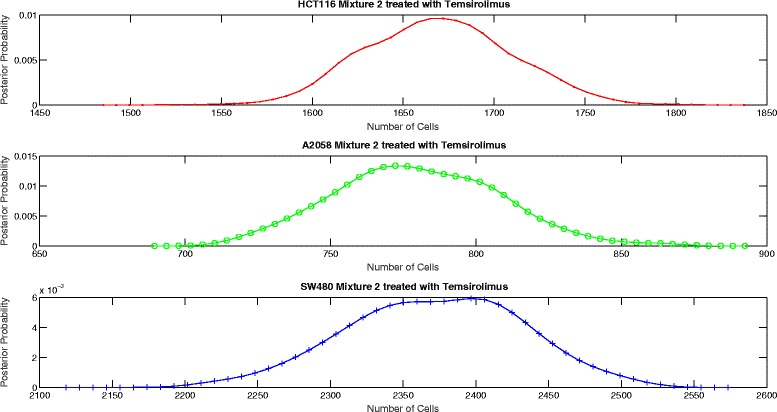
Posterior probability distribution of *N* for mixture 2 treated with Temsirolimus

The proposed aggregate attribute value based algorithm was run for the six test cases. As an input, the algorithm took the summation of the mixture cell-by-cell attribute values as the aggregate input and the single cell line cell-by-cell data for the corresponding group to estimate the mean and the standard deviation of the attributes. The output of the algorithm was the posterior probability of the number of cells for each case as presented in Figs. 8, 9, 10, 11, 12 and 13. The estimate of the number of cells by the algorithm was given by the MAP estimate, represented as $\hat {N}_{agg}$.

Table 2 presents the values of $\hat {N}_{cbc}$ and $\hat {N}_{agg}$ for 6 different experiments. Table 3 shows the corresponding values of *π*_*cbc*_, *π*_*agg*_ and *e*. As it can be observed, the estimate of the number of cells obtained from the algorithm is close to the approximate number of cells obtained by cell-by-cell analysis of the mixture for HCT116 and A2058.

**Table 2:** Number of cells in the mixture obtained by cell-by-cell analysis and aggregate attribute analysis

Experiment	$\hat {N}_{cbc}$	$\hat {N}_{agg}$
Untreated mixture 1	[3314 3710 2070]	[3418 3543 14]
Untreated mixture 2	[1466 757 1557]	[1509 688 1979]
Lapatinib mixture 1	[2440 3812 2060]	[2613 3630 1287]
Lapatinib mixture 2	[1558 691 1782]	[1494 679 2804]
Temsirolimus mixture 1	[2756 3833 1991]	[2794 3855 98]
Temsirolimus mixture 2	[1767 741 1490]	[1668 772 2397]

**Table 3:** Ratios *π*_*cbc*_ and *π*_*agg*_ for cell-by-cell analysis and aggregate attribute analysis respectively and error *e*

Experiment	*π*_*cbc*_	*π*_*agg*_	*e*
Untreated mixture 1	[0.364 0.408 0.228]	[0.490 0.508 0.002]	0.2774
Untreated mixture 2	[0.388 0.200 0.412]	[0.361 0.165 0.474]	0.0761
Lapatinib mixture 1	[0.294 0.459 0.247]	[0.347 0.482 0.171]	0.0955
Lapatinib mixture 2	[0.386 0.171 0.443]	[0.300 0.136 0.564]	0.1525
Temsirolimus mixture 1	[0.321 0.447 0.232]	[0.414 0.571 0.015]	0.2667
Temsirolimus mixture 2	[0.442 0.185 0.373]	[0.344 0.160 0.496]	0.1592

However, the estimate of the number of cells of SW480 is very inaccurate. In particular, we see that the estimate of number of SW480 cells is low for mixture 1 and high for mixture 2. As per the experiment design, the blue fluorescence of all the three cell lines impacts the estimate of number of SW480 cells. Hence, we look at the mean blue attribute value in the single cell line cell-by-cell data and the mixture cell-by-cell data given in Table 4. We observe that for mixture 1, for all the three groups, the mean blue attribute value is less than the mean value for each of the individual cell lines. This accounts for a lower estimate of the number of SW480 cells in mixture 1. Similarly, we see that for mixture 2, the mean value of blue attribute is greater than the mean value for each of the individual cell lines for Lapatinib and Temsirolimus experiments. This accounts for a higher estimate of SW480 cells. For the untreated case for mixture 2, though the mean value of blue attribute is a little lower than the mean attribute value for SW480, it is still on the higher side given that more than half the cells in the mixture are either HCT116 or A2058. However, the discrepancy is not big. As a result the estimate of the number of SW480 cells is on the higher side but not extremely inaccurate.

**Table 4:** Mean Blue Attribute Value for the three cell lines in single cell line cell-by-cell Data, Mixture 1 Cell-by-Cell Data and Mixture 2 Cell-by-Cell Data

Experiment	Cell line	Single	Mixture 1	Mixture 2
Untreated	HCT116	3.013 ×10^6^	2.241 ×10^6^	3.670 ×10^6^
	A2058	3.001 ×10^6^		
	SW480	3.693 ×10^6^		
Lapatinib	HCT116	4.324 ×10^6^	4.215 ×10^6^	6.167 ×10^6^
	A2058	4.620 ×10^6^		
	SW480	5.422 ×10^6^		
Temsirolimus	HCT116	2.670 ×10^6^	2.570 ×10^6^	3.839 ×*S*10^6^
	A2058	3.690 ×10^6^		
	SW480	3.379 ×10^6^		

Hence, by this analysis, we observe that the algorithm performs well if the parameters of the attribute vector remain consistent in the single cell line cell-by-cell analysis and the heterogeneous mixture. Variation in these parameters leads to inaccurate results.

## Discussion

The proposed algorithm enables low cost estimation of the composition of heterogeneous cancer tissue which is an important factor in cancer diagnosis and research. As demonstrated by the simulation results, the algorithm gives an accurate estimate of the different cell lines in the tissue. A crucial aspect of the method proposed is the accurate experiment design. An inconsistent experiment design in the parameter estimation phase and aggregate measurement phase may result in inaccurate estimates of the composition of cell lines as is evident in the experimental results for SW480 cell line. This calls for standardization of the experiment design to ensure the scalability of the algorithm.

## Conclusion

In this work we address the challenge of determining the composition of any heterogeneous cancer tissue. It uses the advantage offered by the expensive cell-by-cell analysis methods while actually utilizing the low cost aggregate attribute methods. The algorithm takes as inputs the characteristics of the attribute vector of the individual cell lines and the aggregate attribute values of the heterogeneous cancer tissue. Based on these inputs, the algorithm uses a Bayesian approach to estimate the number of cells of different cell lines that are present in the heterogeneous mixture. In order to estimate the posterior probability, the algorithm uses the Metropolis algorithm to gather samples from the posterior distribution and Kernel Density Estimation to estimate the distribution from these samples.

## References

[1] Polyak K (2011). Heterogeneity in breast cancer. J Clinical Invest.

[2] Blanco-Calvo M, Concha n, Figueroa A, Garrido F, Valladares-Ayerbes M (2015). Colorectal cancer classification and cell heterogeneity: A systems oncology approach. Int J Mol Sci.

[3] Quail D, Joyce J (2013). Microenvironmental regulation of tumor progression and metastasis. Nat Med.

[4] Marusyk A, Polyak K (2010). Tumor heterogeneity: Causes and consequences. Biochimica et Biophysica Acta (BBA) - Reviews on Cancer.

[5] McGranahan N, Swanton C (2015). Biological and therapeutic impact of intratumor heterogeneity in cancer evolution. Cancer Cell.

[6] Turner NC, Reis-Filho JS (2012). Genetic heterogeneity and cancer drug resistance. Lancet Oncol.

[7] Gatenby RA, Silva AS, Gillies RJ, Frieden BR (2009). Adaptive therapy. Cancer Research.

[8] Sima C, Hua J, Lopes R, Datta A, Bittner ML. Detecting cell growth and drug response in heterogeneous populations: A dynamic imaging approach. In: 2016 IEEE 16th International Conference on Bioinformatics and Bioengineering (BIBE),2016. p. 121–128. 10.1109/BIBE.2016.55.

[9] Mohanty AK, Datta A, Venkatraj V (2014). A model for cancer tissue heterogeneity. IEEE Trans Biomed Eng.

[10] Mohanty AK, Datta A, Venkatraj V (2016). A conjugate exponential model for cancer tissue heterogeneity. IEEE J Biomed Health Informatics.

[11] Hoff PD (2009). A First Course in Bayesian Statistical Methods.

[12] Scott DW (2015). Multivariate Density Estimation: Theory, Practice, and Visualization.

[13] Verhaak RGW, Hoadley KA, Purdom E, Wang V, Qi Y, Wilkerson MD, Miller CR, Ding L, Golub T, Mesirov JP, Alexe G, Lawrence M, O’Kelly M, Tamayo P, Weir BA, Gabriel S, Winckler W, Gupta S, Jakkula L, Feiler HS, Hodgson JG, James CD, Sarkaria JN, Brennan C, Kahn A, Spellman PT, Wilson RK, Speed TP, Gray JW, Meyerson M, Getz G, Perou CM, Hayes DN (2010). Integrated genomic analysis identifies clinically relevant subtypes of glioblastoma characterized by abnormalities in pdgfra, idh1, egfr, and {NF1}. Cancer Cell.

[14] Ellsworth RE, Blackburn HL, Shriver CD, Soon-Shiong P, Ellsworth DL (2017). Molecular heterogeneity in breast cancer: State of the science and implications for patient care. Seminars Cell Dev Biol.

[15] Balgobind BV, Zwaan CM, Pieters R, Van den Heuvel-Eibrink MM (2011). The heterogeneity of pediatric mll-rearranged acute myeloid leukemia. Leukemia.

[16] Macintosh CA, Stower M, Reid N, Maitland NJ (1998). Precise microdissection of human prostate cancers reveals genotypic heterogeneity. Cancer Res.

[17] Gerlinger M, Rowan AJ, Horswell S, Larkin J, Endesfelder D, Gronroos E, Martinez P, Matthews N, Stewart A, Tarpey P, Varela I, Phillimore B, Begum S, McDonald NQ, Butler A, Jones D, Raine K, Latimer C, Santos CR, Nohadani M, Eklund AC, Spencer-Dene B, Clark G, Pickering L, Stamp G, Gore M, Szallasi Z, Downward J, Futreal PA, Swanton Ca (2012). Intratumor heterogeneity and branched evolution revealed by multiregion sequencing. New England J Med.

